# Petrous apex metastasis revealing prostate cancer: a rare case report and review of literature

**DOI:** 10.1097/RC9.0000000000000962

**Published:** 2026-09-09

**Authors:** Marouf Rim, Bijou Walid, Oukessou Youssef, Rouadi Sami, Abada Redallah, Mahtar Mohamed

**Affiliations:** Faculty of Medicine and Pharmacy, Department of Otolaryngology, Head and Neck Surgery, Ibn Rochd University Hospital, Hassan II University, Casablanca, Morocco

**Keywords:** case report, facial nerve palsy, metastasis, otologic symptoms, petrous apex, prostate cancer, skull base, temporal bone

## Abstract

**Introduction and importance:**

Temporal bone malignancies are rare, representing only 0.2% of head and neck malignancies, with an annual incidence of 1 to 6 cases per million. Metastatic involvement of the petrous apex is particularly uncommon, with prostate cancer accounting for approximately 8.6% of temporal bone metastases. We report an extremely rare case of metastatic prostate cancer presenting with otologic symptoms and a destructive petrous apex lesion in a patient with no prior oncological history.

**Case presentation:**

A 73-year-old male presented with right-sided tinnitus, vertigo, and headaches. Audiological workup was unremarkable aside from bilateral presbycusis. CT and magnetic resonance imaging (MRI) imaging revealed a lytic lesion of the right petrous apex extending to the occipital basion and compressing the jugular bulb. Diagnostic mastoidectomy with biopsy demonstrated a poorly differentiated invasive adenocarcinoma with immunohistochemical profile confirming prostatic origin. Prostate-specific antigen was above 100 ng/mL. Positron emission tomography–computed tomography (PET-CT) revealed hypermetabolic prostatic disease with widespread osseous and lymph node metastases. The patient was subsequently initiated on androgen deprivation therapy (ADT) + radiotherapy.

**Discussion:**

Temporal bone metastases from prostate cancer are exceedingly rare, with fewer than 30 cases reported in the literature. Diagnosis is frequently delayed due to nonspecific otologic presentation and the osteolytic pattern, which is atypical for prostatic metastases. A multimodal approach combining high-resolution computed tomography, MRI, prostate-specific membrane antigen PET-CT and histopathological analysis with immunohistochemistry is essential for accurate diagnosis. Treatment remains primarily palliative, incorporating androgen deprivation therapy, radiotherapy, and emerging systemic agents.

**Conclusion:**

A malignant metastatic etiology must always be considered, especially prostate cancer in male patients, even in the absence of a known oncological history. Early recognition through thorough radiological and pathological workup is essential to optimize management and quality of life.

## Introduction

Temporal bone malignancies occupy a rare but clinically consequential niche in head and neck oncology. Representing only 0.2% of all head and neck malignancies, their estimated annual incidence ranges from 1 to 6 cases per million individuals, a rarity that inevitably delays diagnosis and complicates management^[^1^]^.

The temporal bone can serve as an occasional – and frequently underrecognized- site of hematogenous metastasis. Among metastatic deposits to this region, carcinomas of breast and lung origin predominate, with prostate cancer accounting for approximately 8.6% of cases and ranking third overall^[^2,3^]^.

HIGHLIGHTS

- Temporal bone metastases are rare and frequently underdiagnosed due to their nonspecific otologic presentation.
- Metastatic involvement of the petrous apex from prostate cancer is exceedingly rare, with fewer than 30 cases reported in the literature.
- Prostate cancer can initially manifest as isolated skull base involvement, in the absence of any prior oncologic history.
- An osteolytic pattern on imaging, although atypical for prostatic metastases, should not exclude the diagnosis.
- A multimodal workup combining high-resolution computed tomography, magnetic resonance imaging, prostate-specific membrane antigen PET/CT, and immunohistochemistry is essential for accurate diagnosis.
- In any patient with a destructive petrous apex lesion, a malignant metastatic etiology must always be actively considered.

Metastatic involvement of the petrous apex represents the most common subset within this rare entity. Given the deep anatomical position of the petrous apex, lesions in this region present a distinctive diagnostic and surgical challenge. Symptoms are often nonspecific and overlap with far more common otologic and neurological conditions, rendering early recognition difficult and increasing the risk of delayed or erroneous diagnosis^[^1^]^.

In this paper, we present an extremely rare case of metastatic prostate cancer presenting with otologic symptoms and a destructive lesion centered on the petrous apex, occurring in a patient with no prior oncological diagnosis. This case is, to our knowledge, among the very few reported in the literature in which the primary malignancy was identified through investigation of a petrous apex lesion. The purpose of this report is to discuss the clinical and radiological features that should raise suspicion for metastatic disease in this anatomical region, to review the relevant differential diagnoses, and to propose a rational framework for multidisciplinary management in this rare clinical scenario.

This case report has been reported in accordance with the SCARE criteria^[^4^]^.

## Case report

We report the case of a 73-year-old male patient with no known pathological history who presented to our ENT department with right-sided tinnitus of 3 months’ duration, associated with vertigo and headaches, without any other otological symptoms.

Clinical examination revealed a stable patient in good general condition. Vestibular assessment showed no signs of deficits, and otoscopy was unremarkable. Pure-tone audiometry demonstrated bilaterally symmetric presbycusis. Tympanometry revealed a flat curve on the right side, while stapedial reflexes were present. Videonystagmography and video head impulse test were both normal.

A CT scan of the temporal bones identified a lytic lesion of the right temporal bone forming a pseudo-mass extending to the basilar portion of the occipital bone and encompassing the jugular foramina, and magnetic resonance imaging (MRI) confirmed the presence of a lytic process involving the right petrous apex, extending to the occipital basion without intracranial extension, and compressing the jugular bulb (Fig. 1).

**Figure 1.**
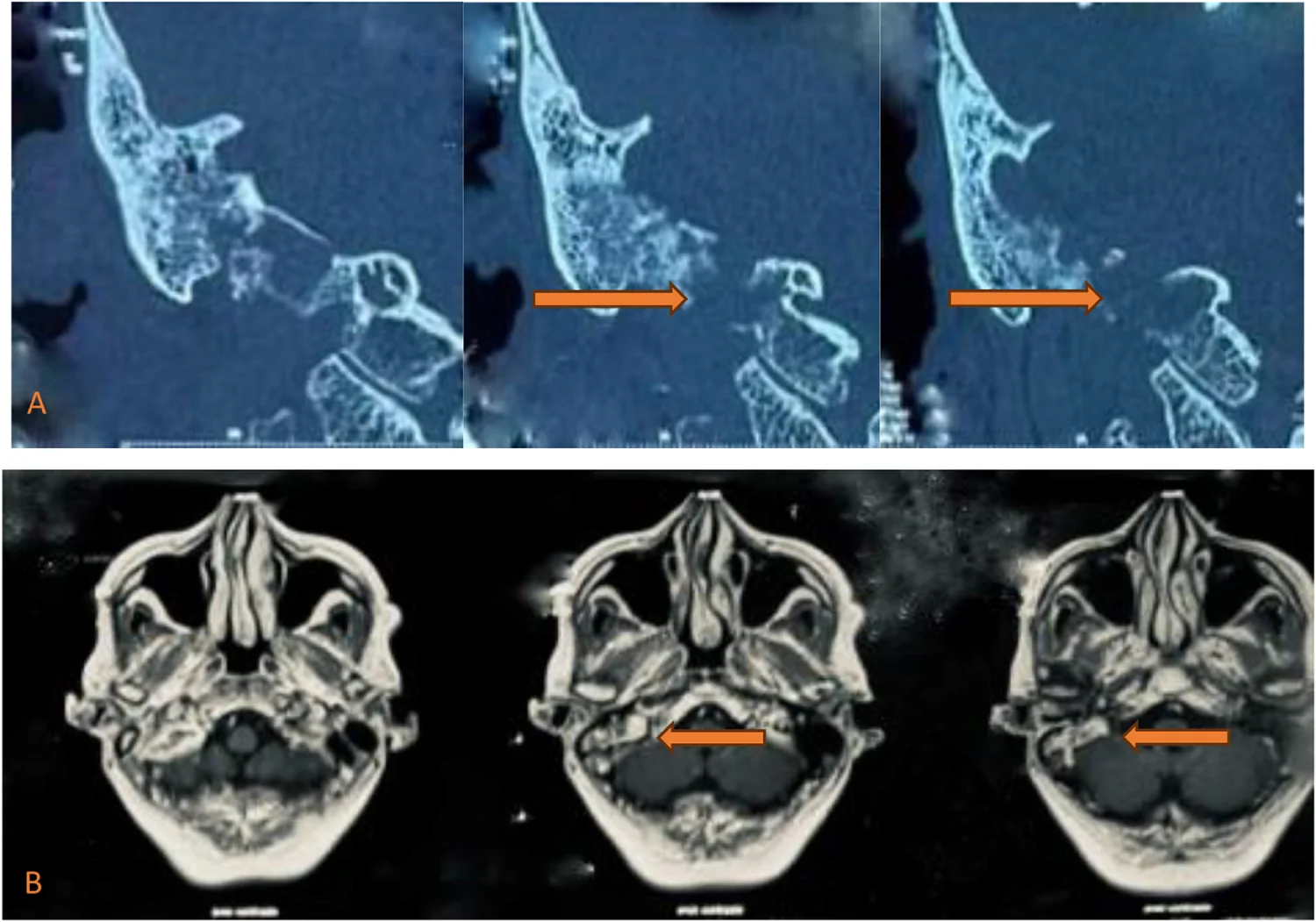
CT scan of the right temporal bone (A) and cranial MRI images (B) showing a lytic process involving the right petrous apex and extending to the occipital basion. MRI, magnetic resonance imaging.

Based on these findings, a diagnostic mastoidectomy with biopsy was performed. Histopathological examination revealed osseous involvement by a poorly differentiated invasive adenocarcinoma. Complementary immunohistochemical analysis showed that the infiltrate expressed racemase and prostate-specific antigen (PSA) and was negative for both CK7 and CK20, confirming osseous metastasis from prostatic adenocarcinoma (Fig. 2).

**Figure 2.**
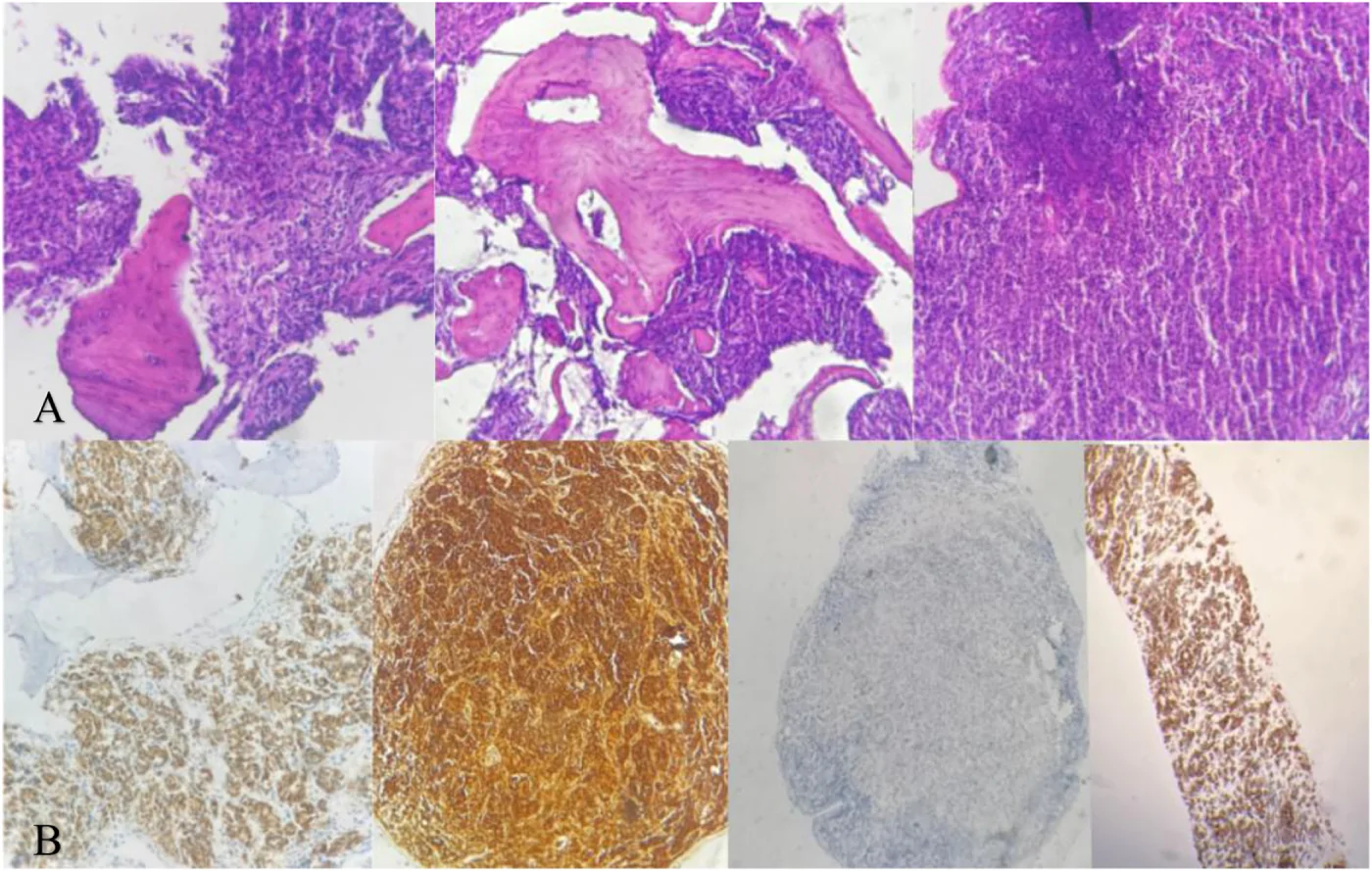
(A) Histological images demonstrating a carcinomatous proliferation with extensive crush artifact, infiltrating the adjacent bone with focally glandular architecture. (B) Immunohistochemistry shows strong and diffuse expression of CKAE1/AE3 and racemase, nuclear positivity for PSA, and negative CK7 and CK20 expression.

A prostatic biopsy was performed, confirming the diagnosis, with a PSA above 100 ng/ml. The patient subsequently underwent positron emission tomography–computed tomography scanning, which demonstrated a hypermetabolic prostate with multiple hypermetabolic osseous foci involving the skull, spine, rib cage, scapula, sternum, pelvis, and right femur, as well as diffuse lymph node involvement. The patient is currently undergoing hormone therapy and radiotherapy (Fig. 3).

**Figure 3.**
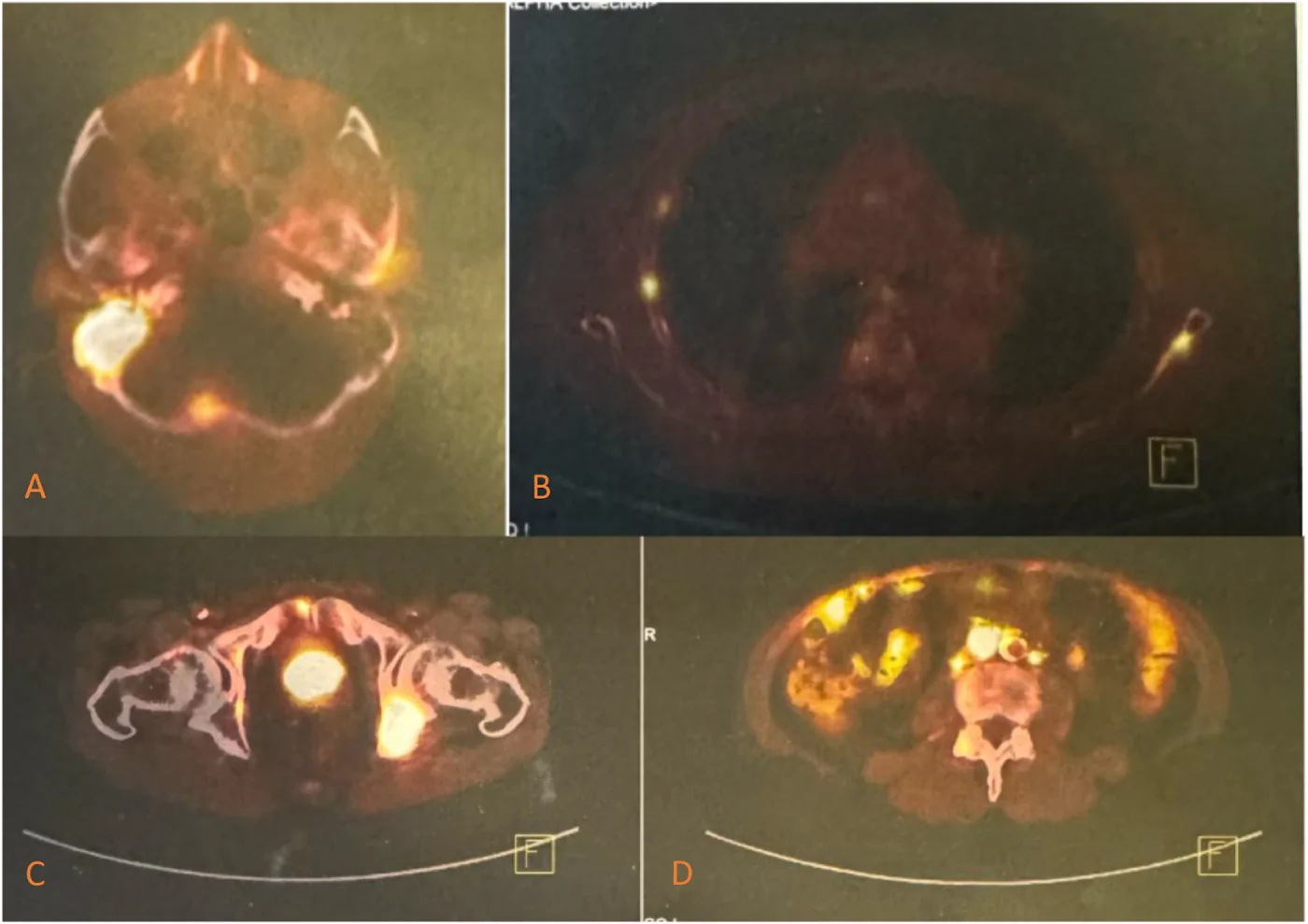
PET-CT images showing hypermetabolic osseous foci in the right temporal bone (A), rib cage (B), and prostate and pelvis (C), as well as diffuse lymph node involvement (D). PET-CT, positron emission tomography–computed tomography.

Given that a meaningful assessment of the clinical course and treatment response requires extended observation, longer-term follow-up data will be reported in a future update once sufficient time has elapsed.

## Discussion

Prostate cancer (PCa) is known for its potential for bone metastases, especially in the axial skeleton. However, temporal bone metastasis remains rare, accounting for 8% according to the latest review, with 21 cases reported in the literature as of 2015, and fewer than 9 additional individual cases reported since that time^[^1,5,6^]^.

The most common pathway for tumor spread is hematogenous. While the petrous apex, mastoid, and internal auditory canal are all possible sites of tumor spread, the petrous apex is the most common. This is likely due to its rich blood supply from Batson’s venous plexus, which connects the prostate to the skull through epidural and dural veins^[^6^]^.

Many cases are asymptomatic (40%) and are found only at autopsy. In other cases, it may present with hearing loss, peri-auricular swelling, otorrhea, otalgia, vertigo, and tinnitus. Some rare manifestations of reported metastases include multiple cranial nerve palsies, ophthalmoplegia, and isolated facial nerve palsy. It can even closely mimic primary brain tumors or subdural hematoma^[^2,3,7–11^]^.

Radiological imaging is crucial for diagnosis. High-resolution computed tomography is superior to MRI for determining bone involvement and skull base destruction, due to its high-resolution bone algorithms. Skeletal metastases due to prostate cancer are more commonly osteoblastic than osteolytic. However, the osteolytic type appears to be the most common in temporal bone metastases, which was the scenario in our case. MRI is more sensitive in detecting leptomeningeal, dural, or cranial nerve involvement and is thus complementary to CT^[^3,12,13^]^.

prostate-specific membrane antigen (PSMA) PET/CT is important for localization, characterization, and follow-up of the disease. It can detect sites of disease recurrence at lower PSA levels than conventional imaging, especially in castration-resistant cases. Serum PSA is the most common biomarker for screening and for detecting disease recurrence after initial treatment. Levels typically rise with metastasis and correlate with tumor volume^[^6,14^]^.

The differential diagnosis of a destructive petrous apex is broad and can be organized into three main categories: benign/pseudotumoral entities, primary neoplasms, and malignant/metastatic disease.

Benign and pseudotumoral lesions include cholesterol granuloma, the most common petrous apex lesion overall, typically presenting with expansile, well-circumscribed changes rather than frank destruction; petrous apicitis, usually associated with a clinical context of otomastoiditis and Gradenigo syndrome; and congenital cholesteatoma, which characteristically shows a scalloped, expansile pattern of bone erosion.

Malignant and metastatic lesions should be strongly considered when imaging demonstrates permeative, ill-defined bone destruction without a discernible matrix. This category includes primary temporal bone carcinoma, nasopharyngeal carcinoma with skull base extension, and hematogenous metastases. Among metastatic primaries, lung, breast, and renal carcinomas are the most frequently implicated, while prostate carcinoma (as in the present case) should be specifically considered in older male patients, and its presentation can closely mimic primary skull base disease, underscoring the importance of a thorough systemic oncological workup whenever imaging raises suspicion for a destructive petrous apex process, particularly in the absence of a known primary malignancy^[^2,3,15^]^.

Pathological examination remains essential for both diagnosis and prognosis. PCa bone metastases can be divided into two groups according to androgen receptor (AR) activity: high or low AR activity. These two groups of bone metastases have different immune cell profiles. Moreover, following genome-wide association studies (GWAS) of PCa bone metastases, three distinct molecular subtypes have been identified: MetA, MetB, and MetC, each having different gene expression, morphology, and clinical features. MetA is the most frequent and has high expression of AR-regulated genes, including PSA. MetB shows poor prognosis after androgen deprivation therapy (ADT) and has some characteristics similar to neuroendocrine tumors. It exhibits profiles related to DNA damage and cell cycle activity, with androgen-stimulated gene expression generally low and cell proliferation high. MetC shows high transcriptional activity and is the least common and most poorly defined. Using PSA and Ki67 analysis, MetA-like and MetB-like tumors can be differentiated, each with a different prognosis. High expression of Ki67 in combination with low PSA expression and a low fraction of AR-positive stroma correlates with poor prognosis after ADT and with the development of the MetB subtype. The opposite pattern predicts the development of the MetA subtype with a better ADT response^[^16–18^]^.

Treatment remains primarily palliative. Radium-223-dichloride is a bone-targeting agent approved for patients with symptomatic bone metastases from PCa without visceral disease. For metastatic hormone-sensitive PCa, several studies have demonstrated that the addition of abiraterone to androgen-deprivation therapy improves overall survival. Surgery is usually required for pathological diagnosis and may be used for debulking in symptomatic patients^[^19,20^]^.

Prognosis is poor for patients with metastatic cancer to the temporal bone, with an average survival time after diagnosis of less than 2 years. Patients presenting with cranial nerve palsies typically survive less than 6 months. In recent decades, while alternative treatments such as taxane chemotherapy, androgen receptor pathway inhibitor, radium-223 dichloride, and 177 Lu-labeled prostate-specific membrane antigen (177Lu-PSMA) have demonstrated acceptable benefits for overall survival in metastatic CRPC (mCRPC), novel therapeutic strategies and interventions are continuously being developed and applied to more effectively control the progression and metastasis risk of PCa based on molecular mechanisms^[^21,22^]^.

The main strength of this report is the rarity and educational value of petrous apex metastasis as the initial presentation of previously undiagnosed prostate cancer. However, as a single case report, our findings do not allow for generalizable conclusions, and the limited available follow-up limits the assessment of long-term outcomes.

## Conclusion

Metastatic disease of the temporal bone, and the petrous apex in particular, remains a diagnostically challenging entity whose rarity should not lead to its exclusion from the differential diagnosis. In any patient presenting with unexplained hearing loss, facial nerve palsy, or otologic symptoms refractory to conventional treatment, a malignant etiology must be actively considered. As illustrated by this case, prostate cancer can manifest initially through isolated skull base involvement, mimicking benign or inflammatory conditions and resulting in significant diagnostic delay.

While the prognosis of temporal bone metastases remains guarded, early recognition offers the opportunity to optimize systemic treatment, provide meaningful palliation, and improve quality of life.

### Patient perspective

The patient reported that the initial symptoms prompting medical evaluation were distressing and unexpected, and he expressed concern regarding the uncertain diagnosis during the initial diagnostic workup. Following further investigation and multidisciplinary discussion, he was reassured by the clarity provided through imaging and histopathologic confirmation, which allowed a clear therapeutic plan to be established. He expressed satisfaction with the coordinated care provided by the otolaryngology, radiology, and oncology teams and appreciated being informed and involved in decisions regarding his management.

## Data Availability

The data that support the findings of this study are available from the corresponding author upon reasonable request.
